# The Cleansing of Root Canals

**Published:** 1912-01-15

**Authors:** A. W. Ward

**Affiliations:** San Francisco, Cal.


### THE CLEANSING OF ROOT CANALS.

BY A. W. WARD, D.D.S., SAN FRANCISCO, CAL.

You will please excuse me if I take up five minutes of your valuable time with a very trite subject, cleansing of root canals. The only excuse I can offer is that I have found something which I believe is a great aid in this work. When peroxide of hydrogen was first introduced it was acknowledged that without its aid it was impossible to remove all the contents of the canals.

In the treatment of canals that have been previously treated with any drug that coagulates albumen, peroxide of hydrogen fails to give the desired results. You have all perhaps noticed this to be the case. Sulphuric acid has been and still is a standby with the majority of the profession, because of its dehydrating property, nevertheless it is far from a perfect means to the end, because of its great coagulating effect.

To make myself plain, suppose one treats a putrescent tooth with sulphuric acid, does he not form a wall down the sides of the canal, which may block in bacteria that will be impossible of access by means of our after-treatment? Still another fault of the sulphuric acid is that it disorganizes the structure of the dentine. We have all noticed, in teeth treated with this drug, how abnormally white the floor of the pulp chamber becomes, and how readily it can be scooped away with an excavator. This proves that the dental structure has been destroyed by the action of the acid on the mineral salts. Will not this structure be an admirable medium for the subsequent lodgement of pathological microorganisms?

Sodium dioxide has been recommended as an opener for intricate canals. The same objection applies to it that applies to sulphuric acid. Sodium and potassium have also been used, but in my experience one of these is about as good as the other. The Sodium compounds are supposed to act upon the fatty contents of the canals, saponifying them and converting them into a substance that is easily removed.

Gentlemen, my little offering is soap, pure and unadulterated. The soap I am now using is the preparation known as Ethereal Soap. Let me enumerate some of its virtues as an opener and cleanser.

First, we will suppose that a fresh pulp has been removed by means of pressure anesthesia. It is good practice to swab out the canals with carbolic acid to cauterize any remaining fibers that may be clinging to the walls of the canals. In doing this we of course coagulate the blood that is nearly always present, and at a subsequent sitting find it very difficult to remove the coagulum, as peroxide of hydrogen will not dissolve it, neither will sulphuric acid.

In a case of this kind, if the canals are swabbed out with the Ethereal Antiseptic Soap and afterward with peroxide of hydrogen, the results will astonish you.

Second, in reaming out the canals we do not need anything to dissolve the contents; we need a lubricant.

Did you ever notice a man sawing a large bar of iron? If you have, you probably observed that now and then he ran his saw through a bar of soap. When you next have some small canals to open, try a little soap. Fill the pulp chamber and carefully work through with a smooth broach followed by Kerr broaches.

Sometimes we are called upon to treat a tooth the canals of which are filled with a cheesy substance which is very foul. It takes a great many treatments, sometimes, to render them free from odor, even without the grand new tri-cresol-formaline treatment. The number of treatments can be greatly dimished, if, after the first treatment, soap is used as an aid in the removal of the contents of the canals.

I suppose I have used more than my alloted five minutes, but please allow me to quote an article from a recent *Cosmos* which will probably set you to thinking more along this line:

"Soap may be classified with the familiar antiseptics, its microbial actions having been proven beyond doubt by numerous experiments. Its action is neither toxic nor irritant, and is not impaired by fatty substances, which on the contrary it removes, thus preparing the tissues for the influence of other drugs. Soap generally should not be mixed with other antiseptics, especially not with sublimate, as it counteracts its effects. Formalin, however, can be advantageously incorporated into soap."

As a further argument let me remind you that soap has been used for a long time in surgery to cleanse open wounds, to cleanse the hands before operating, in obstetrics, etc.

